# Targeting DNA topoisomerases: past & future

**DOI:** 10.20517/cdr.2021.65

**Published:** 2021-07-21

**Authors:** William H. Gmeiner, Robert C. A. M. van Waardenburg

**Affiliations:** ^1^Department of Cancer Biology Wake Forest School of Medicine, Medical Center Blvd, Winston-Salem, NC 27157, USA.; ^2^Department of Pharmacology and Toxicology, University of Alabama at Birmingham, Birmingham, AL 35294-0019, USA.

Aggressive malignancies are characterized by relatively uncontrolled cell proliferation making them especially reliant on topoisomerase enzymes to enable high rates of DNA replication and transcription. DNA topoisomerases resolve topological problems associated with DNA replication and other essential cellular processes involving DNA, such as transcription and recombination^[1]^. As such, they are important targets for anti-cancer therapeutics. Further, understanding of topoisomerase biology is important for unraveling the mechanistic basis for resistance to many widely used anti-cancer drugs, such as doxorubicin, etoposide, and topotecan, for which DNA topoisomerases are established targets. Interestingly, several drugs that are not considered to directly target topoisomerase enzymes, such as the nucleoside analogs 5-FU and AraC, and those in development such as the polymeric fluoropyrimidine CF10^[2]^, also affect the function of topoisomerases.

DNA topoisomerase II (Top2) enzymes reduce DNA superhelicity by introducing a transient DNA double strand break to facilitate transport of another region of duplex DNA through the break. The Top2α isoform is only expressed during G2-M phase, and it is a major target for anticancer drugs, including etoposide and doxorubicin that interfere in a re-ligation step of the Top2α catalytic cycle, stabilizing the Top2α cleavage complex (Top2cc), and converting the enzyme into a cellular poison. The activity of DNA Top2α is modulated by multiple post-translational modifications (PTMs) including phosphorylation, acetylation, ubiquitination, and SUMOylation. A characteristic of cancer cells is increased or otherwise altered PTMs modulating enzyme activity, and Top2α is no exception. In this issue, Lotz and Lamour^[3]^ reviewed the interplay between Top2α PTMs and drug resistance. Top2α activity is modulated by multiple phosphorylation sites and cancer cells phosphorylate Top2α at a number of sites not identified in non-malignant cells including the conserved catalytic tyrosine (Y805), which is phosphorylated in Jurkat cells and K562 acute leukemia cells. Further systematic analysis of Top2α PTM will be required to define their role in drug resistance and to develop new therapeutic approaches targeting Top2α.

Several of the most effective and widely used anti-cancer therapeutics target Top2 and understanding the basis for drug resistance to these is of particular concern. Etoposide and doxorubicin are Top2 poisons that trap Top2cc resulting in persistent DNA double strand breaks that can cause cancer cells to undergo programmed cell death. The activity of Top2 poisons requires expression of the enzyme and its nuclear localization. Cells deficient in Top2α expression or nuclear localization are relatively resistant to Top2 poisons. Elton *et al.*^[4]^ have identified alternative splice variants of the Top2α protein that do not undergo nuclear localization and cells expressing these variants are resistant to Top2α poisons. In this issue, Elton *et al.*^[4]^ review Top2α splice variants and the role of alternative splicing as a cause of drug resistance that predominates in some cancer cell lines. Alternative splicing is an important mechanism affecting drug activity that demands consideration in addition to drug efflux mechanisms.

Human DNA topoisomerase I (Top1) is a Type IB topoisomerase that relieves both positive and negative supercoils in DNA generated during replication and transcription. In this issue, Soren *et al.*^[5]^ review the mechanism of human Top1 and detail how this mechanism is targeted by specific anti-cancer drugs. Top1 releases topological stress via a controlled rotation mechanism in which a reactive tyrosine (Y723) nicks the DNA by forming a transient covalent cleavage complex (Top1cc). Relaxation of DNA supercoiling then proceeds spontaneously within the Top1 C-type clamp that circumscribes the enzyme-bound and free DNA ends. The enzyme subsequently catalyzes a second transesterification reaction to restore the integrity of the DNA double helix. As is the case for Top2α, compounds that reversibly stabilize Top1cc are termed Top1 poisons since these result in persistent DNA double-strand breaks that cause cancer cells to undergo apoptosis. Top1 poisons bind neither the DNA substrate nor the enzyme in its apo state, rather they are specific for the cleavage complex and Soren *et al.*^[5]^ describe the structural basis for Top1 poisoning and distinguish this mechanism from inhibitors of enzymatic activity.

Camptothecin (CPT) is a natural product identified in an anti-cancer screen by Wall *et al.*^[6]^ in 1966 and shown to be a Top1 poison. Synthetic analogs of CPTs (e.g., irinotecan, topotecan) are widely used anti-cancer drugs. Pommier and co-workers showed Top1 poisoning also occurs via oxidative base damage and as a result of DNA binding by carcinogens (e.g., benzo[a]pyrene). Subsequent studies extended these findings to demonstrate that nucleoside analogs such as cytarabine inhibit the re-ligation step of Top1 catalysis and cause DNA DSBs. Further, the importance of Top1 poisoning was demonstrated by reduced activity in Top1-deficient cells. In this issue, Gmeiner^[7]^ reviews the poisoning of Top1 by nucleoside analogs including recent findings that the Top1cc formed by nucleoside analogs shows different dependence on repair enzymes, such as Tdp1, indicating that Top1 poisoning by nucleoside analogs is a distinct and complementary process to the poisoning by CPTs.

The potential danger that the hallmark formation of a transient enzyme-DNA covalent complex by DNA topoisomerases form to release the topological stress that occurs from pulling apart the double-stranded DNA helix for DNA synthesis and transcription to occur, seemed to be a calculated risk by nature. Since the discovery of DNA topoisomerases by Wang^[8]^ in 1971, and the revelation of their catalytic mechanism, the field anticipated that nature developed a “solution” to the formation of the potentially toxic Top1cc or Top2cc. Scientists proposed an enzyme that was able to hydrolyze the phosphodiester linkage formed between the topoisomerases active site tyrosine and the end of a DNA strand. It was not until 1996 when Yang *et al.*^[9]^ from the NIH reported this hallmark enzyme activity with the discovery of Tyrosyl-DNA phosphodiesterase I (Tdp1) in yeast. The review of Brettrager and van Waardenburg^[10]^ in this issue focuses on Tdp1 and its potential as an anti-cancer target. Brettrager and van Waardenburg^[10]^ highlight the Tdp1 catalytic mechanism to remove Top1 from its covalent complex with the DNA. Removal of Top1 from its Top1-DNA complex occurs via a transient Tdp1-DNA covalent complex (Tdp1cc) thus forming a potential toxic enzyme-DNA adduct itself. Currently two different approaches are developed to use this surprising Achilles’ heel in the fight against cancer. The first one is to inhibit Tdp1 enzyme activity, thus preventing Tdp1 from removing the drug stabilized Top1cc or Top2cc. Small molecule Tdp1 inhibitors are being developed by various groups throughout the world as discussed in this review. A second potential approach to target Tdp1 for the treatment of cancer was emphasized with the discovery that dysregulation of Tdp1 catalytic mechanism via the substitution of one of the two Tdp1 catalytic histidines to an arginine is the molecular basis for the rare autosomal neurodegenerative disease, SCAN1. This mutation of the general acid/base His to Arg resulted in an increased half-life of Tdp1cc that is detrimental for cerebellar cells. This suggests that identification of small molecules that stabilize the Tdp1cc similar to the Top1 and Top2 poisons would offer a novel therapeutic strategy in the fight against cancer.

Over the last 50 years, we have gained a wealth of knowledge on maintenance of DNA topology by DNA topoisomerases, and how we can use their mechanism of action to treat human disease, specifically cancer. In this special issue, recent highlights in our understanding of DNA topoisomerase I and II biology, use of topoisomerase-targeting therapeutics, and the mechanism of repair of enzyme-DNA reaction intermediates are summarized. Although, topoisomerase-targeting therapeutics are highly successful in the clinic, novel therapeutic approaches are urgently needed to circumvent drug-resistance mechanisms and to treat additional cancer types. Moreover, development of small molecules to target DNA topoisomerase III would complement and expand the current anti-cancer treatment options directed at topoisomerases. As such, it is clear that there is much more knowledge to be gained to understand the role of DNA topoisomerases in human disease and more effectively treat cancer patients and overcome resistance to anti-cancer drugs.

## DECLARATIONS

### Copyright

© The Author(s) 2021.
